# Extended LTA, TNF, LST1 and HLA Gene Haplotypes and Their Association with Rubella Vaccine-Induced Immunity

**DOI:** 10.1371/journal.pone.0011806

**Published:** 2010-07-27

**Authors:** Inna G. Ovsyannikova, Robert A. Vierkant, V. Shane Pankratz, Robert M. Jacobson, Gregory A. Poland

**Affiliations:** 1 Vaccine Research Group, Mayo Clinic, Rochester, Minnesota, United States of America; 2 Department of Pediatric and Adolescent Medicine, Mayo Clinic, Rochester, Minnesota, United States of America; 3 Department of Health Sciences Research, Mayo Clinic, Rochester, Minnesota, United States of America; 4 Program in Translational Immunovirology and Biodefense, Mayo Clinic, Rochester, Minnesota, United States of America; Lund University, Sweden

## Abstract

**Background:**

Recent studies have suggested the importance of HLA genes in determining immune responses following rubella vaccine. The telomeric class III region of the HLA complex harbors several genes, including lymphotoxin alpha (LTA), tumor necrosis factor (TNF) and leukocyte specific transcript -1 (LST1) genes, located between the class I B and class II DRB1 loci. Apart from HLA, little is known about the effect of this extended genetic region on HLA haplotypic backgrounds as applied to immune responses.

**Methodology/Principal Findings:**

We examined the association between immune responses and extended class I-class II-class III haplotypes among 714 healthy children after two doses of rubella vaccination. These extended haplotypes were then compared to the HLA-only haplotypes. The most significant association was observed between haplotypes extending across the HLA class I region, ten-SNP haplotypes, and the HLA class II region (i.e. A-C-B-LTA-TNF-LST1-DRB1-DQA1-DQB1-DPA1-DPB1) and rubella-specific antibodies (global *p*-value of 0.03). Associations were found between both extended A*02-C*03-B*15-AAAACGGGGC-DRB1*04-DQA1*03-DQB1*03-DPA1*01-DPB1*04 (*p* = 0.002) and HLA-only A*02-C*03-B*15-DRB1*04-DQA1*03-DQB1*03-DPA1*01-DPB1*04 haplotypes (*p* = 0.009) and higher levels of rubella antibodies. The class II HLA-only haplotype DRB1*13-DQA1*01-DQB1*06-DPA1*01-DPB1*04 (*p* = 0.04) lacking LTA-TNF-LST1 SNPs was associated with lower rubella antibody responses. Similarly, the class I-class II HLA-only A*01-C*07-B*08-DRB1*03-DQA1*05-DQB1*02-DPA1*01-DPB1*04 haplotype was associated with increased TNF-α secretion levels (*p* = 0.009). In contrast, the extended AAAACGGGGC-DRB1*01-DQA1*01-DQB1*05-DPA1*01-DPB1*04 (*p* = 0.01) haplotype was found to trend with decreased rubella-specific IL-6 secretion levels.

**Conclusions/Significance:**

These data suggest the importance of examining both HLA genes and genes in the class III region as part of the extended haplotypes useful in understanding genomic drivers regulating immune responses to rubella vaccine.

## Introduction

Studies conducted by others and ourselves have supported the importance of HLA genes in determining both humoral and cellular immune responses to rubella vaccine. For example, the HLA class II genes, and particularly the DRB1*04-DQB1*03-DPB1*03 and DRB1*15/16-DQB1*06-DPB1*03 haplotypes significantly influence rubella-specific antibody responses [1]. Evidence continues to accumulate however, that the immune response to viral vaccines is controlled by more than just HLA-specific polymorphisms. In fact, a large and growing family of non-HLA genes has recently been identified as critical to the immune response to vaccination, including rubella [2]–[4]. Recent studies support the idea that polymorphisms in immune-response candidate-genes should be analyzed as haplotypes rather than as individual alleles based on evidence for multigenic control of immune responses to infectious pathogens [5]–[7].

Several genes encoded in the class III region of the HLA complex on chromosome 6 (6p21.3) - including lymphotoxin alpha (LTA), tumor necrosis factor (TNF) and leukocyte specific transcript -1 (LST1) genes – are linked between the HLA-B and HLA-DRB1 loci [8]. Because of their linkage to HLA class I and II genes, it has been suggested that TNF and/or LTA (and LST1) may contribute to combinations or haplotypes of allelic variants that differ in composition and occurrence, and may contribute to the etiology of HLA-associated infectious diseases and immunity [5], [8], [9]. Studies have associated specific single nucleotide polymorphisms (SNPs) in either the TNF or LTA locus, along with HLA-B and/or HLA-DRB1 loci, with severe dengue virus infection, malaria, and susceptibility to autoimmune diseases [5], [6], [10]; however, extended LTA, TNF, LST1, and class I and class II HLA haplotype associations with rubella vaccine-induced immune responses have not been examined.

TNF and LTA are proinflammatory cytokines with important biological activities and immunomodulatory functions and are known to influence a variety of cellular responses [11]. Polymorphisms in the genes encoding TNF and LTA appear to contribute to infectious disease susceptibility and infection [12]–[16]. The leukocyte specific transcript-1 is a gene with extensive alternative splicing and is encoded within the TNF region of the HLA complex, and plays a role in inflammatory and infectious diseases [17], [18]. The LST1 gene is constitutively expressed in PBMCs and has an inhibitory effect on lymphocyte proliferation [17]. Hence, LTA, TNF, LST1, and additional genes encoded in the telomeric class III HLA region are potentially important genetic markers of vaccine-induced immunity. We sought to test the hypothesis that inclusion of class III genes in extended class I-class II gene haplotypes provides information beyond HLA alone. For this reason we investigated associations between rubella-specific antibody and cytokine levels with 33 haplotypes containing polymorphisms for LTA, TNF, and LST1 candidate genes. We analyzed extended SNP-defined LTA, TNF, LST1, and HLA haplotype profiles and examined their contribution to humoral and cellular immune responses following the recommended two doses of the US licensed rubella-containing vaccine. This builds upon the work on HLA-only haplotype associations that we previously reported [1], [19], [20].

## Results

### Associations between Extended Haplotypes and Rubella-Specific Antibodies

As previously reported, we genotyped 714 healthy adolescents and young adults (336 females and 378 males) following two doses of rubella vaccine [21]. Summaries of demographic and clinical characteristics for these subjects can be found in **Table 1**. The median rubella-specific antibody level in our study cohort was 34.5 (IQR 19.2–63.7) IU/ml. We previously examined associations between HLA haplotypes and rubella antibody levels and found that class I A*01-C*07-B*07 (*p* = 0.04) and A*03-C*07-B*07 (*p* = 0.04) haplotypes were associated with higher and lower rubella-specific IgG antibodies, respectively [20]. Further, we found that the class II DRB1*04-DQB1*03-DPB1*03 (*p* = 0.01) and DRB1*15/16-DQB1*06-DPB1*03 (*p* = 0.005) haplotypes were associated with lower levels of IgG antibodies [20].

**Table 1:** Demographic characteristics of the study population (n = 714).

Variable	Category	No. of subjects (%)
Overall		714
Age at enrollment	11–13 years	212 (29.7%)
	14–15 years	190 (26.6%)
	16–17 yeas	200 (28.0%)
	18–19 yeas	112 (15.7%)
Age at first rubella vaccination	< = 14 months	89 (12.5%)
	15 months	384 (53.8%)
	16–17 months	119 (16.6%)
	> = 18 months	122 (17.1%)
Age at second rubella vaccination	< = 5 years	205 (28.7%)
	6–10 years	109 (15.3%)
	11 years	122 (17.1%)
	> = 12 years	278 (38.9%)
Gender	Female	336 (47.1%)
	Male	378 (52.9%)
Race	Other	65 (9.1%)
	White	649 (90.9%)
Cohort	First	324 (45.4%)
	Second	390 (54.6%)

**Figure 1:**
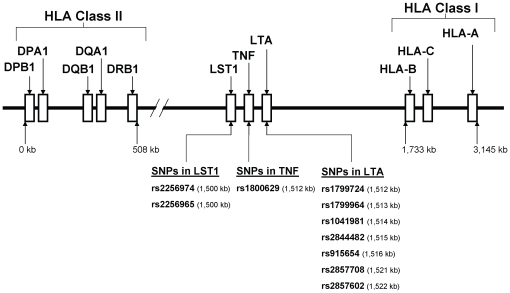
A schematic overview of the extended human leukocyte antigen (HLA) complex, encompassing the lymphotoxin alpha (LTA), tumor necrosis factor (TNF) and leukocyte specific transcript -1 (LST1) genes on chromosome 6 (6p21.3). A 3,145 kb segment of the extended HLA gene region is shown. The LTA, TNF, and LST1 loci encoded in the class III region of the HLA complex in relation to HLA-B and HLA-DRB1 loci. The positions of single nucleotide polymorphisms (SNPs) selected from LTA [rs2857602 (31641357), rs2857708 (31641585), rs915654 (31646476), rs2844482 (31647746), rs1041981 (31648763), rs1799964 (31650287), rs1799724 (31650461)], TNF [rs1800629 (31651010)], and LST1 [rs2256965 (31663109), rs2256974 (31663371)] gene families and used for haplotype estimation are shown. Targeted sets of extended haplotypes used in this study: (1) haplotypes containing the three class I HLA loci (A-C-B) and the set of ten-SNP haplotypes, (2) haplotypes containing ten-SNP haplotypes and the class II HLA loci (DRB1-DQA1-DQB1-DPA1-DPB1), and (3) haplotypes containing class I HLA loci (A-C-B), the set of ten-SNP haplotypes, and alleles of class II HLA loci (DRB1-DQA1-DQB1-DPA1-DPB1). Note: the figure is not to scale.

Testing the hypothesis that our further extended haplotypes provide information beyond the HLA haplotypes, separate analyses were performed for each of three sets of combinations or extended haplotypes: one containing the three class I HLA loci (A-C-B) and the set of ten-SNP haplotypes, one containing ten-SNP haplotypes and the class II HLA loci (DRB1-DQA1-DQB1-DPA1-DPB1), and one containing class I HLA loci (A-C-B), the set of ten-SNP haplotypes, and the class II HLA loci (DRB1-DQA1-DQB1-DPA1-DPB1) (**Figure 1**). To examine our hypothesis, these sets of extended haplotypes were compared to the HLA-only haplotypes.

A total of 12 extended haplotypes with frequencies of **≥**1% were identified in the first set of allelic combinations. **Table 2** shows specific haplotypes with significant (*p*<0.05) or suggestive (*p*≤0.15) associations with rubella IgG antibody levels. The global test for association failed to show a statistically significant association between haplotypes extending across the class I HLA region and the set of ten-SNP haplotypes and rubella antibody levels (*p*-value of 0.29). While this difference is not statistically significant, given the lack of global significance, several observations are of potential interest. When examining extended HLA class I, LTA, TNF, and LST1 haplotypes individually, the A*02-C*03-B*15-AAAACGGGGC(test statistic of 2.54, *p* = 0.01) and A*03-C*07-B*07-GGTGCAGGAC (test statistic of −2.34, *p* = 0.02) haplotypes trended with higher and lower rubella humoral antibody responses, respectively. Among these individually statistically significant extended haplotypes, the class I HLA-only haplotype A*03-C*07-B*07 (test statistic of -2.60, *p* = 0.01) was also associated with lower rubella antibodies, indicating that inclusion of LTA-TNF-LST1 SNPs did not substantially change the strength of this genetic association. By contrast, the HLA-only haplotype A*02-C*03-B*15 lacking SNPs demonstrated no association with rubella-specific antibodies (*p* = 0.15).

**Table 2:** Associations between extended haplotypes and HLA-only haplotypes, and rubella virus-specific antibody responses.

Class, haplotype	Haplotype^b^	Haplotype frequency	Test statistic (haplotype t-statistic)	Allele p-value^a^	Global p-value	HLA-only haplotype frequency	Test statistic (haplotype t-statistic)	Allele p-value^a^	Global p-value
A-C-B-RS2857602-RS2857708-RS915654-RS2844482-RS1041981-RS1799964-RS1799724-RS1800629-RS2256965-RS2256974									
A-C-B-LTA-TNF-LST1					0.288				0.159
	*02*03*15-AAAACGGGGC	0.026	2.54	**0.011**		0.039	1.44	0.151	
	03*07*07-GGTGCAGGAC	0.050	−2.34	**0.020**		0.056	−2.60	**0.010**	
RS2857602-RS2857708-RS915654-RS2844482-RS1041981-RS1799964-RS1799724-RS1800629-RS2256965-RS2256974-DRB1-DQA1-DQB1-DPA1-DPB1									
LTA-TNF-LST1-DRB1-DQA1-DQB1-DPA1-DPB1					0.107				**0.035**
	AGAGAAGGGA-*13*01*06*01*04	0.012	−1.46	0.146		0.042	−2.07	**0.039**	
	AAAACGGGGC-*01*01*05*01*04	0.022	1.68	0.093		0.055	0.64	0.522	
	AAAACGGGGC-*04*03*03*01*04	0.024	2.31	**0.021**		0.100	1.66	0.098	
A-C-B-RS2857602-RS2857708-RS915654-RS2844482-RS1041981-RS1799964-RS1799724-RS1800629-RS2256965-RS2256974-DRB1-DQA1-DQB1-DPA1-DPB1									
A-C-B-LTA-TNF-LST1- DRB1-DQA1-DQB1-DPA1-DPB1					**0.033**				0.168
	*02*03*15-AAAACGGGGC-*04*03*03*01*04	0.013	3.16	**0.002**		0.018	2.62	**0.009**	

Only haplotypes with estimated frequencies ≥0.01 and *p*-values ≤0.15 are presented.

a Haplotype effects estimated using a haplotype t-statistic that assesses associations of antibody levels across haplotypes. Allele *p*-value compares the haplotype of interest to all other haplotypes combined. Statistically significant findings (*p*<0.05) are shown in bold. Analyses adjust for age at blood draw, gender, race, age at first rubella vaccine, age at second rubella vaccine, and cohort status.

b Common SNPs from the LTA gene: [rs2857602 (A>G), rs2857708 (G>A), rs915654 (T>A), rs2844482 (G>A), rs1041981 (C>A), rs1799964 (A>G), rs1799724 (G>A)]; TNF gene: rs1800629 (G>A); LST1 gene: [rs2256965 (G>A), and rs2256974 (C>A)].

A total of 15 extended haplotypes with frequencies of **≥**1% were identified in the second set of allelic combinations. For the extended LTA, TNF, LST1, and HLA class II region haplotypes, the global test did not reach statistical significance (*p*-value of 0.11). The individual haplotype with the strongest numerical value for association with higher rubella-specific antibody response was AAAACGGGGC-DRB1*04-DQA1*03-DQB1*03-DPA1*01-DPB1*04 (test statistic of 2.31, *p* = 0.02). We also found trends for lower levels of rubella-specific antibody marginally associated with the AAAACGGGGC-*01*01*05*01*04 haplotype (test statistic of 1.68, *p* = 0.09). For HLA-only haplotypes and rubella antibodies, the global *p*-value was 0.03. Associations were significant for the class II HLA-only haplotype DRB1*13-DQA1*01-DQB1*06-DPA1*01-DPB1*04 (test statistic of −2.07, *p* = 0.04), while neither DRB1*04-DQA1*03-DQB1*03-DPA1*01-DPB1*04 (*p* = 0.10) nor *01*01*05*01*04 (*p* = 0.5) showed any trend.

A total of 6 extended haplotypes with frequencies of **≥**1% were identified in the third set of allelic combinations. The most significant association was observed between these haplotypes extending across the HLA class I region, ten-SNP haplotypes, and the HLA class II region (i.e. A-C-B-LTA-TNF-LST1-DRB1-DQA1-DQB1-DPA1-DPB1) and rubella virus-specific antibodies (global *p*-value of 0.03). We found haplotypes associated with very high humoral immune response among rubella-vaccinated individuals. The specific haplotype with the strongest association was A*02-C*03-B*15-AAAACGGGGC-DRB1*04-DQA1*03-DQB1*03-DPA1*01-DPB1*04 (test statistic of 3.16, *p* = 0.002). Similarly, the HLA-only A*02-C*03-B*15-DRB1*04-DQA1*03-DQB1*03-DPA1*01-DPB1*04 haplotype (test statistic of 2.62, *p* = 0.009) was significantly associated with high rubella humoral immune response (**Table 2**). These associations were slightly stronger when including the set of ten-SNP haplotypes.

### Associations between Extended HLA class I, LTA, TNF, LST1 Haplotypes and Rubella-Specific Cytokines

We previously examined associations of HLA haplotypes with rubella-specific cytokine production and found that several class I haplotypes were potentially associated with variations in cytokine secretion levels [19]. In our study the overall cytokine profile was skewed towards a predominant Th1/inflammatory response as previously described [21]. Median values (IQR) for rubella-specific IFN-γ, IL-2, IL-6, TNF-α, and GM-CSF cytokine secretion levels were 8.5 (3.0, 23.4) pg/ml, 17.6 (7.7, 30.5) pg/ml, 3681.0 (3160.0, 4052.0) pg/ml, 29.7 (−7.0, 89.2) pg/ml, and 28.0 (23.6, 32.6) pg/ml, respectively. A total of 12 haplotypes extending across the class I region (i.e. A-C-B-LTA-TNF-LST1) with frequencies of **≥**1% were identified. **Tables 3** **–** [Table pone-0011806-t004] **5** only report haplotypes with significant (*p*<0.05) or suggestive (*p*≤0.15) associations with rubella virus-specific secreted cytokines.

**Table 3:** Associations between extended class I-HLA-LTA-TNF-LST1 haplotypes and class I HLA-only haplotypes, and rubella-specific cytokine responses.

Cytokine	Haplotype^b^	Haplotype frequency	Test statistic (haplotype t-statistic)	Allele p-value^a^	Global p-value	HLA-only haplotype frequency	Test statistic (haplotype t-statistic)	Allele p-value^a^	Global p-value
A-C-B-RS2857602-RS2857708-RS915654-RS2844482-RS1041981-RS1799964-RS1799724-RS1800629-RS2256965-RS2256974									
IFN-γ					0.759				0.345
	*03*04*35-AGTGCAGGAC	0.015	1.58	0.115		0.016	2.32	**0.020**	
IL-2					0.235				0.299
	*02*03*15-AAAACGGGGC	0.026	1.65	0.100		0.039	0.61	0.545	
	*02*07*07-GGTGCAGGAC	0.035	−2.12	**0.035**		0.034	−1.45	0.149	
IL-6					0.617				0.239
	*01*06*57-AGTGCGGGGC	0.012	−2.01	**0.045**		0.012	−1.95	0.052	
	*24*07*07-GGTGCAGGAC	0.016	1.49	0.137		0.018	2.64	**0.009**	
TNF-α					0.835				0.310
	*01*07*08-AGTGAAGAGC	0.080	1.58	0.114		0.086	1.44	0.151	
	*29*16*44-GGTGCAGGAC	0.022	2.30	**0.022**		0.023	2.35	**0.019**	
GM-CSF					0.888				0.744
	*02*07*07-GGTGCAGGAC	0.035	1.60	0.109		0.034	0.91	0.365	

Only haplotypes with estimated frequencies ≥0.01 and *p*-values ≤0.15 are presented.

a Haplotype effects estimated using a haplotype t-statistic that assesses associations of cytokine levels across haplotypes. Allele *p*-value compares the haplotype of interest to all other haplotypes combined. Statistically significant findings (*p*<0.05) are shown in bold. Analyses adjust for age at blood draw, gender, race, age at first rubella vaccine, age at second rubella vaccine, and cohort status.

b Common SNPs from the LTA gene: [rs2857602 (A>G), rs2857708 (G>A), rs915654 (T>A), rs2844482 (G>A), rs1041981 (C>A), rs1799964 (A>G), rs1799724 (G>A)]; TNF gene: rs1800629 (G>A); LST1 gene: [rs2256965 (G>A), and rs2256974 (C>A)].

**Table 4 pone-0011806-t004:** Associations between extended LTA-TNF-LST1-class II HLA haplotypes and class II HLA-only haplotypes, and rubella-specific cytokine responses.

Cytokine	Haplotype^b^	Haplotype frequency	Test statistic (haplotype t-statistic)	Allele p-value^a^	Global p-value	HLA-only haplotype frequency	Test statistic (haplotype t-statistic)	Allele p-value^a^	Global p-value
RS2857602-RS2857708-RS915654-RS2844482-RS1041981-RS1799964-RS1799724-RS1800629-RS2256965-RS2256974-DRB1-DQA1-DQB1-DPA1-DPB1									
IFN-γ					0.409				0.615
	AGTGCAGGAC-*01*01*05*01*04	0.014	1.63	0.103		0.055	0.50	0.620	
	AGTGAAGAGC-*15*01*06*01*04	0.011	−2.01	**0.044**		0.097	−0.35	0.723	
	AGAGAAGGGA-*13*01*06*01*04	0.012	−2.73	**0.006**		0.042	−1.83	0.068	
	GGTGCAGGAC-*04*03*03*01*04	0.023	2.09	**0.037**		0.100	1.13	0.259	
IL-2					0.135				0.151
	AGAGAAGGGA- *04*03*03*01*04	0.038	2.49	0.013		0.100	2.87	**0.004**	
	AGAGAAGGGA-*13*01*06*01*04	0.012	−1.56	0.118		0.042	−1.37	0.172	
	AAAACGGGGC-*04*03*03*01*04	0.024	1.84	0.066		0.100	2.87	**0.004**	
	GGTGCAGGAC-*07*02*02*01*04	0.023	1.95	0.051		0.027	1.86	0.064	
IL-6					0.053				0.816
	AGTGAAGAGC-*03*05*02*01*04	0.042	−1.83	0.068		0.049	−2.07	**0.039**	
	AGTGAAGAGC-*15*01*06*01*04	0.011	1.95	0.052		0.097	0.53	0.597	
	AGAGAAGGGA-*13*01*06*01*04	0.012	1.64	0.101		0.042	0.72	0.469	
	AAAACGGGGC-*01*01*05*01*04	0.022	−2.53	**0.012**		0.055	−1.38	0.169	
TNF-α					0.610				0.446
	AGTGCGGGGC-*07*02*03*01*04	0.013	−1.64	0.101		0.014	−1.86	0.064	
GM-CSF					0.642				0.910
	AAAACGGGGC-*01*01*05*01*04	0.022	−2.09	**0.037**		0.055	−0.53	0.597	

Only haplotypes with estimated frequencies ≥0.01 and *p*-values ≤0.15 are presented.

a Haplotype effects estimated using a haplotype t-statistic that assesses associations of cytokine levels across haplotypes. Allele *p*-value compares the haplotype of interest to all other haplotypes combined. Statistically significant findings (*p*<0.05) are shown in bold. Analyses adjust for age at blood draw, gender, race, age at first rubella vaccine, age at second rubella vaccine, and cohort status.

b Common SNPs from the LTA gene: [rs2857602 (A>G), rs2857708 (G>A), rs915654 (T>A), rs2844482 (G>A), rs1041981 (C>A), rs1799964 (A>G), rs1799724 (G>A)]; TNF gene: rs1800629 (G>A); LST1 gene: [rs2256965 (G>A), and rs2256974 (C>A)].

**Table 5:** Associations between extended class I HLA-LTA-TNF-LST1-class II HLA haplotypes and class I-class II HLA-only haplotypes, and rubella-specific cytokine responses.

Cytokine	Haplotype^b^	Haplotype frequency	Test statistic (haplotype t-statistic)	Allele p-value^a^	Global p-value	HLA-only haplotype frequency	Test statistic (haplotype t-statistic)	Allele p-value^a^	Global p-value
A-C-B-RS2857602-RS2857708-RS915654-RS2844482-RS1041981-RS1799964-RS1799724-RS1800629-RS2256965-RS2256974-DRB1-DQA1-DQB1-DPA1-DPB1									
IFN-γ					0.597				0.242
	*02*07*07-GGTGCAGGAC-*15*01*06*01*04	0.019	1.56	0.120		0.019	2.26	**0.024**	
	*03*07*07-GGTGCAGGAC-*15*01*06*01*04	0.032	−1.73	0.084		0.034	−2.57	**0.010**	
IL-2					0.446				0.209
	*02*07*07-GGTGCAGGAC-*15*01*06*01*04	0.019	−1.52	0.129		0.019	−1.80	0.073	
IL-6					0.965				0.943
TNF-α					0.059				0.042
	*01*07*08-AGTGAAGAGC-*03*05*02*01*04	0.038	2.53	**0.012**		0.038	2.64	**0.009**	
GM-CSF					0.382				0.445
	*01*07*08-AGTGAAGAGC-*03*05*02*01*04	0.038	1.55	0.123		0.038	1.65	0.100	

Only haplotypes with estimated frequencies ≥0.01 and *p*-values ≤0.15 are presented.

a Haplotype effects estimated using a haplotype t-statistic that assesses associations of cytokine levels across haplotypes. Allele *p*-value compares the haplotype of interest to all other haplotypes combined. Statistically significant findings (*p*<0.05) are shown in bold. Analyses adjust for age at blood draw, gender, race, age at first rubella vaccine, age at second rubella vaccine, and cohort status.

b Common SNPs from the LTA gene: [rs2857602 (A>G), rs2857708 (G>A), rs915654 (T>A), rs2844482 (G>A), rs1041981 (C>A), rs1799964 (A>G), rs1799724 (G>A)]; TNF gene: rs1800629 (G>A); LST1 gene: [rs2256965 (G>A), and rs2256974 (C>A)].

Separate analyses were performed for every measure of cytokine response (IFN-γ, IL-2, IL-6, TNF-α, and GM-CSF). The global tests for association failed to show a statistically significant association between allelic combinations and rubella-specific cytokine responses measured by ELISA (**Table 3**). When examining haplotypes individually, the class I extended A*02-C*07-B*07-GGTGCAGGAC (t-statistic of −2.12, *p* = 0.03) and A*01-C*06-B*57-AGTGCGGGGC(t-statistic of −2.01, *p* = 0.04) haplotypes were suggestive of lower rubella-specific IL-2 and IL-6 cytokine responses, respectively.

We found no associations between class I HLA-only haplotypes and lower rubella-specific cytokine responses. On the contrary, we found trends for higher levels of IFN-γ and IL-6 associated with class I HLA-only haplotypes such as A*03-C*04-B*35 (t-statistic of 2.32, *p* = 0.02) and A*24-C*07-B*07 (t-statistic of 2.64, *p* = 0.009), respectively. We found no evidence that LTA-TNF-LST1 alleles alter the strengths of the genetic associations between extended haplotypes and rubella-specific IFN-γ and IL-6.

We also found trends for higher levels of the inflammatory cytokine, TNF-α, associated with both the extended haplotype A*29-C*16-B*44-GGTGCAGGA (t-statistic of 2.30, *p* = 0.02) and the HLA-only haplotype A*29-C*16-B*44 (t-statistic of 2.35, *p* = 0.02).

However, these findings should be interpreted with caution, due to the lack of a significant global test result.

### Associations between Extended LTA, TNF, LST1, HLA Class II Haplotypes and Rubella-Specific Cytokines

We previously also examined associations of HLA haplotypes with rubella-specific cytokine production and found that several class II haplotypes were potentially associated with variations in cytokine secretion levels [19]. In this study a total of 15 extended haplotypes (i.e. LTA-TNF-LST1-DRB1-DQA1-DQB1-DPA1-DPB1) with frequencies of **≥**1% were identified. The five global tests failed to show a statistically significant association between SNPs in LTA, TNF and LST1 genes and across the class II HLA haplotypes and rubella-specific IFN-γ, IL-2, TNF-α, and GM-CSF, although for the haplotypes and IL-6 secretion the global *p*-value was suggestive but not statistically significant (*p* = 0.05) (**Table 4**).

The extended AAAACGGGGC-DRB1*01-DQA1*01-DQB1*05-DPA1*01-DPB1*04 haplotype (t-statistic of −2.53, *p* = 0.01) was observed to trend with decreased rubella-specific IL-6 secretion levels. We also found trends for lower levels of IL-6 secretion associated with the class II HLA-only DRB1*03-DQA1*05-DQB1*02-DPA1*01-DPB1*04 haplotype (t-statistic of −2.07, *p* = 0.04). In contrast, the AGTGAAGAGC-DRB1*15-DQA1*01-DQB1*06-DPA1*01-DPB1*04 haplotype (t-statistic of 1.95, *p* = 0.05) was found to trend with increased IL-6 secretion levels.

We also found trends for higher levels of IFN-γ secretion associated with the extended GGTGCAGGAC-DRB1*04-DQA1*03-DQB1*03-DPA1*01-DPB1*04 haplotype (t-statistic of 2.09, *p* = 0.04). On the contrary, two extended AGTGAAGAGC-DRB1*15-DQA1*01-DQB1*06-DPA1*01-DPB1*04 (t-statistic of −2.01, *p* = 0.04) and AGAGAAGGGA-DRB1*13-DQA1*01-DQB1*06-DPA1*01-DPB1*04 (t-statistic of −2.73, *p* = 0.006) haplotypes were observed to be significantly associated with decreased rubella-specific IFN-γ secretion levels. We found no associations between class II HLA-only haplotypes and variations in rubella-specific IFN-γ cytokine responses. In addition, the individual extended haplotype with statistical evidence of a support for trend toward an association with higher IL-2 responses was AGAGAAGGGA-DRB1*04-DQA1*03-DQB1*03-DPA1*01-DPB1*04 (t-statistic of 2.49, *p* = 0.01). The class II HLA-only DRB1*04-DQA1*03-DQB1*03-DPA1*01-DPB1*04 haplotype (t-statistic of 2.87, *p* = 0.004) was also observed to be significantly associated with increased rubella-specific IL-2 secretion levels, suggesting that LTA-TNF-LST1 SNPs are not contributing to the effect of IL-2 response associations.

Finally, we also found trends for lower levels of inflammatory GM-CSF with the extended AAAACGGGGC- DRB1*01-DQA1*01-DQB1*05-DPA1*01-DPB1*04 haplotype (t-statistic of −2.09, *p* = 0.04). However, these associations must be considered preliminary given the absence of a significant global test result. Neither rubella-specific GM-CSF secretion nor TNF-α secretion levels were associated with class II HLA-only haplotypes.

### Associations between Extended HLA Class I, LTA, TNF, LST1, HLA Class II Haplotypes and Rubella-Specific Cytokines

A total of 6 haplotypes extending across the class I and II regions (i.e. A-C-B-LTA-TNF-LST1-DRB1-DQA1-DQB1-DPA1-DPB1) with frequencies of **≥**1% were identified. Associations between HLA class I and class II haplotypic backgrounds with LTA-TNF-LST1 SNPs and rubella-specific cytokine immune responses are presented in **Table 5** **.** Global tests failed to find significant associations between extended haplotypes and rubella-specific TNF-α secretion (*p*-value of 0.06). However, the A*01-C*07-B*08-AGTGAAGAGC-DRB1*03-DQA1*05-DQB1*02-DPA1*01-DPB1*04 haplotype (t-statistic of 2.53, *p* = 0.01) was found to be associated with increased TNF-α secretion levels. Conversely, the global tests suggested associations between TNF-α secretion and class I-class II HLA-only haplotypes (*p*-value of 0.04). Specifically, the A*01-C*07-B*08-DRB1*03-DQA1*05-DQB1*02-DPA1*01-DPB1*04 haplotype (t-statistic of 2.64, *p* = 0.009) was significantly associated with increased TNF-α secretion levels. This suggests that these associations were mostly driven by HLA gene polymorphisms and not by the LTA-TNF-LST1 alleles *per se*.

The global tests failed to provide evidence of statistically significant associations between extended haplotypes and IFN-γ, IL-2, IL-6, and GM-CSF production. However, individual HLA-only haplotypes associated with lower and higher rubella-specific IFN-γ responses included A*03-C*07-B*07-DRB1*15-DQA1*01-DQB1*06-DPA1*01-DPB1*04 (t-statistic of -2.57, *p* = 0.01) and A*02-C*07-B*07-DRB1*15-DQA1*01-DQB1*06-DPA1*01-DPB1*04 (t-statistic of 2.26, *p* = 0.02), respectively. No significant associations were detected between IFN-γ, IL-2, and GM-CSF production and any haplotype extending across the class I and II regions.

## Discussion

The HLA genes significantly impact an individual's ability to respond immunologically to rubella vaccine antigens because binding of antigenic peptides occurs within the HLA peptide binding groove, and the repertoire of bound peptides depends on HLA allele-specific polymorphisms [22], [23]. However, HLA gene polymorphisms alone do not explain the entire vaccine-induced immune response. Consistent with the evidence away from a single dominant allele model for complex immune responses, and toward a multigenic network model [24], this study was undertaken to further explore the extent of genetic polymorphisms within the LTA, TNF, LST1, and HLA regions and to expand our understanding of immunogenetic mechanisms of rubella vaccine-induced immunity across a larger genetic region of interest. We hypothesized that the gene products encoded in the HLA region on chromosome 6 operate jointly with other genes to determine rubella vaccine immune response outcomes. A total of 33 unique LTA-TNF-LST1-HLA haplotypes were identified. Class I and class II HLA haplotype associations between antibody, cytokine levels, lymphocyte proliferative, and ELISPOT responses following rubella vaccine have been described [1], [19], [20]. We studied additional genetic markers (SNPs) included in HLA haplotypes, including ten-SNP common polymorphisms, located between HLA-B and HLA-DRB1 loci. The specific extended haplotype with the strongest association with higher humoral immune response among rubella-vaccinated subjects was A*02-C*03-B*15-AAAACGGGGC-DRB1*04-DQA1*03-DQB1*03-DPA1*01-DPB1*04 (*p* = 0.002). Similarly, the HLA-only haplotype A*02-C*03-B*15-DRB1*04-DQA1*03-DQB1*03-DPA1*01-DPB1*04 (*p* = 0.009) was also associated with higher humoral immune response. Importantly, the relative comparison of global tests and individual p-values between haplotypes demonstrated slightly stronger associations when including the set of ten-SNP haplotypes.

This particular extended haplotype may be considered a highly significant immune (antibody) response haplotype to rubella virus vaccine. Additionally, two extended (A*02-C*03-B*15-AAAACGGGGC, *p* = 0.01 and AAAACGGGGC-DRB1*04-DQA1*03-DQB1*03-DPA1*01-DPB1*04, *p* = 0.02) haplotypes trended with higher rubella antibody responses. Effects attributable to these haplotypes were due in part to LTA-TNF-LST1 SNPs, because the HLA-only haplotype DRB1*04-DQA1*03-DQB1*03-DPA1*01-DPB1*04 (*p* = 0.098) showed only a marginal association, while the A*02-C*03-B*15 (*p* = 0.15) haplotype demonstrated no trend. In contrast, the A*03-C*07-B*07-GGTGCAGGAC haplotype (*p* = 0.02) trended with a weaker rubella antibody response. Likewise, the HLA-only A*03-C*07-B*07 haplotype (*p* = 0.01) was associated with a higher humoral immune response. Thus, inclusion of polymorphisms for LTA, TNF, and LST1 candidate genes into this specific HLA haplotype did not alter the strength of the association. This outcome is in agreement with our recent study demonstrating the association of A*03-C*07-B*07 and DRB1*04-DQB1*03-DPB1*04 haplotypes with rubella-specific antibodies [20] along with other earlier studies demonstrating DR3 and DR4 restricted T-cell and B-cell responses to rubella virus antigens [25], as well as defined class I A*3 restricted immune responses to rubella [26]. Nepom *et al*. [27] also demonstrated HLA-DR3 and DR4 restriction of rubella E1 peptides and commented that such analyses “provide a direct approach for selecting antigenic peptides useful for epitope-based vaccines targeted to multiple HLA types”. Our results imply that several genes in the class III region are involved in regulating humoral immune responses to rubella vaccine, and that some signature gene haplotypes can potentially be used to characterize the spectrum of rubella-specific IgG secretion. Further studies need to be performed in larger and other racially diverse populations to more fully investigate the role of these genetic haplotypes (and potential causal variants) in vaccine-induced humoral immune responses.

Since cytokines are important for the development and shaping of innate and adaptive immune responses to rubella, we examined associations between extended haplotypes and rubella virus-specific cytokine (IFN-γ, IL-2, IL-6, TNF-α, and GM-CSF) secretion levels. Interestingly, for the Th1 cytokine IFN-γ, we found evidence for lower levels of IFN-γ secretion associated with the extended LTA-TNF-LST1-DRB1-DQA1-DQB1-DPA1-DPB1 haplotypes. In particular, the haplotype AGTGAAGAGC-DRB1*15-DQA1*01-DQB1*06-DPA1*01-DPB1*04 (*p* = 0.04) was found to trend with decreased rubella-specific IFN-γ secretion levels, while the AGAGAAGGGA-DRB1*13-DQA1*01-DQB1*06-DPA1*01-DPB1*04 haplotype was significantly associated with lower IFN-γ response (*p* = 0.006); however, the global test did not reach statistical significance, which may be a statistical power issue resolvable with larger cohort sizes. When all class II HLA-only haplotypes were compared across cytokine immune responses, we found no HLA haplotypes associated with IFN-γ response. These results imply that genetic variation in the telomeric class III region of the HLA may play a role in modulating IFN-γ immune responses to rubella and are worthy of further investigation.

The haplotypic background DRB1*04-DQA1*03-DQB1*03-DPA1*01-DPB1*04 revealed suggestive associations between both higher IFN-γ (GGTGCAGGAC-DRB1*04-DQA1*03-DQB1*03-DPA1*01-DPB1*04, *p* = 0.04) and higher IL-2 (AGAGAAGGGA-DRB1*04-DQA1*03-DQB1*03-DPA1*01-DPB1*04, *p* = 0.01) rubella-specific secretion. In contrast, class II HLA-only DRB1*04-DQA1*03-DQB1*03-DPA1*01-DPB1*04 (*p* = 0.004) haplotype was observed to be associated with increased rubella-specific IL-2 secretion levels, suggesting that LTA-TNF-LST1 SNPs are not strongly contributing to the effect of IL-2 response associations. We also found potential associations with lower rubella virus-specific IL-2 secretion on the class I haplotypic background A*02-C*07-B*07. Specifically, the extended A*02-C*07-B*07-GGTGCAGGAChaplotype (*p* = 0.03) was found to be associated with lower IL-2 secretion. No associations were found between class I HLA-only haplotypes and rubella-specific IL-2 responses.

We found novel potential associations with the class I A*01-C*07-B*08 and class II DRB1*03-DQA1*05-DQB1*02-DPA1*01-DPB1*04 haplotype containing a set of ten-SNPs and the inflammatory cytokine TNF-α. Specifically, haplotypes A*01-C*07-B*08-AGTGAAGAGC(p = 0.01) and A*01-C*07-B*08-AGTGAAGAGC-DRB1*03-DQA1*05-DQB1*02-DPA1*01-DPB1*04 (*p* = 0.01) were associated with increased rubella-specific TNF-α secretion, though the class I-class II HLA-only A*01-C*07-B*08-DRB1*03-DQA1*05-DQB1*02-DPA1*01-DPB1*04 (*p* = 0.009) haplotype was observed to be significantly associated with increased rubella-specific TNF-α secretion levels. Furthermore, individual class I and class II HLA-only haplotypes demonstrated trends with lower and higher rubella-specific IFN-γ responses; A*03-C*07-B*07-DRB1*15-DQA1*01-DQB1*06-DPA1*01-DPB1*04 (*p* = 0.01) and A*02-C*07-B*07-DRB1*15-DQA1*01-DQB1*06-DPA1*01-DPB1*04 (*p* = 0.02), respectively. Thus, this association was mostly driven by HLA gene polymorphisms and not by the LTA-TNF-LST1 SNPs *per se*. These results are very consistent with the important roles that both HLA and cytokines play in the rubella immunity.

Because the class III region of the HLA is characterized by strong and extended linkage disequilibrium (LD), these findings probably reflect the underlying LD patterns on these haplotypes [28]. The LD pattern could be very diverse on specific DRB1-DQA1-DQB1-DPA1-DPB1 haplotypes. It is also possible that other genes in LD with the HLA genes on chromosome 6 are important in influencing vaccine immune responses.

It should be noted that we have examined a reasonably large collection of potential associations in this work, including three different classes of extended haplotypes (HLA class I plus SNPs, HLA class II plus SNPs, HLA class I and II combined plus SNPs) and six distinct outcomes. We have attempted to apply one level of control to these comparisons by performing a global test of significance for all haplotypes of a given class for each outcome. By performing this first-pass assessment, we are able to control false positives for any given outcome by considering significant only those haplotype associations whose global test is also significant. However, additional care should be exercised given that 18 global tests were performed. Using a 0.05 level of significance, on average there should be 0.9 significant global tests that reach significance, and we observed a single global *p*-value that reached that level of significance. However, 1.8 global tests would be expected to be significant at the 0.1 level of significance while we observed a total of 3 – nearly twice the expected. We identified a total of 33 extended haplotypes with frequencies greater than or equal to 0.01∶12 from HLA class I, 15 from HLA class II, and 6 from HLA class I and II combined. Ignoring the global tests and proceeding with haplotype-specific analyses for each of the six outcomes would have resulted in a total of 198 tests of association. It is important to note that after controlling for multiple testing using a Bonferroni correction (a conservative testing approach due to the correlational structure of both the outcomes and the haplotypes), none of the global or haplotype-specific tests remained significant at the *p* = 0.05 level.

While it is possible that some of the results reported here are false positives, there is a real chance that we have not identified all of the potentially true results. For instance, with a total of 714 subjects providing the data to test the association between an outcome and 15 haplotypes formed from one set of loci, there is 80% power to detect associations if the haplotypes describe 2.7%, or 2.2% of the variability in the outcome if the type I error level is set at 0.05 or 0.10. These are rather large effect sizes; for instance, a haplotype with a frequency of 2% would need to be associated with a greater than 2-fold difference in antibody levels in order to describe an association of this magnitude. Therefore, while there is a potential for false positives among those identified here, it is also possible that important true associations could be missed. There were also associations that were found in extended haplotypes but not in HLA-only haplotypes and vice versa. In addition, the percent of variability in the immune response measures explained by the haplotypes was modest, even for the two models with statistically significant global tests (r-squared = 0.80% for the association of antibody levels with the combined class I and class II haplotype, and 4.98% for the class II only extended haplotype). This is likely to be typical for multi-allelic models of complex phenotypic traits such as immune responses. For all these reasons, the results described herein should be considered discovery in nature. It is important that further studies be performed to replicate, and possibly extend, the findings reported here.

We propose to test the hypothesis that non-HLA variants contribute information above and beyond the HLA associations and that inclusion of class III genes in extended class I-class II gene haplotypes provides additional information beyond HLA alone. We have examined associations between variations in an extended genomic region surrounding the HLA complex and have observed associations between haplotypes spanning the region flanked by the class I and class II loci and rubella vaccine immunity. Importantly, we found significant associations between higher rubella antibody levels and SNPs located within the class I-class II loci on the A*02-C*03-B*15-DRB1*04-DQA1*03-DQB1*03-DPA1*01-DPB1*04 haplotypic background. It is possible that this haplotype pattern marks other (untyped) polymorphic variants or regions in LD, such as HLA-B associated transcripts (BAT3, BAT2 and BAT1), allograft inhibitory factor 1 (AIF-1), natural cytotoxicity triggering receptor (NCR3), lymphotoxin beta (LTB), the MHC class I chain-related gene A (MICA), nuclear factor of κB inhibitor-like polypeptide 1 (NFKBIL 1), complement component C4 and/or others. As genetic variants may function in a complementary manner to determine the outcome of vaccine-induced immune responses, it is logical to propose that the observed antibody level effects in our study may be an outcome of combinations of HLA/SNP-defined alleles, including multigenic or complementation effects. Additional population-based replication studies of larger size are needed in order to assess the association of antibody variations in individuals with this haplotype diversity. Potential extended haplotype associations with variations in rubella vaccine-induced cytokine secreted levels in our study population may also implicate an immunological role of the proteins encoded by the HLA (A-C-B)-LTA-TNF-LST1-HLA (DRB1-DQA1-DQB1-DPA1-DPB1) gene cluster in immune response to rubella vaccine. Genetically determined variations in cytokine secretion levels could influence both innate and adaptive immunity to rubella. Future studies should concentrate on functional characterization of these and other replicated gene products with respect to immunological roles. We plan on confirming these haplotypes in a larger independent replication cohort to validate the findings and to elucidate the functional aspects of these genetic variants.

Our study further demonstrates that HLA genes play a dominant role in immune responses to rubella vaccine. Our study may also suggest the existence of additional immune response candidate genetic polymorphisms situated in the extended HLA region, in addition to the known HLA allelic haplotypes, that may play important roles in regulating immune responses. This new information as well as future studies will help increase our understanding of the genetic factors underlying rubella vaccine-induced immunity and could be useful in the design of new rubella vaccines. Understanding the basis for immune response genotype/phenotype associations further informs knowledge regarding vaccine immune response heterogeneity, and allows the development of directed methods for personalized vaccines [24], [29].

## Materials and Methods

### Ethics Statement

This study complied with the human experimentation guidelines of the United States Department of Health and Human Services, and all enrolled subjects or their parents provided written, informed consent, as well as written assent by age-appropriate children. The work presented here was approved by the Institutional Review Board at the Mayo Clinic under Protocol number 06-002783.

### Study Subjects

As described elsewhere, between 2001 and 2002, we enrolled 346 healthy adolescents (age 12 to 18 years) in Rochester, Minnesota [30]. Three hundred and forty-two parents agreed to allow their adolescents from this study to take part in a second rubella vaccine study (first cohort). From 2006 to 2007, we enrolled another cohort of 396 healthy adolescents and young adults (age 11 to 19 years) in Rochester, Minnesota (second cohort). All 738 participants (combined cohort) had documentation of having received two doses of measles-mumps-rubella-II (MMR-II) vaccine containing the attenuated RA27/3 Wistar strain of rubella virus (Merck) [20]. No known circulating rubella virus was observed since the earliest year of birth for any subject in our geographic area. While 738 children and young adults were enrolled in the study, genotyping data for SNPs and HLA alleles were available for only 714 subjects (**Table 1**).

### Antibody Measurement

We assayed all subjects for quantitative levels of rubella virus-specific IgG antibodies using a whole virus rubella-specific chemiluminescent immunoassay (Beckman Coulter Access, Fullerton, CA) according to the manufacturer's instructions. The limit of detection for this assay was 0.5 IU/ml and the coefficient of variation of this assay in our laboratory was 6%.

### Genotyping Methods

DNA was extracted from fresh heparinized blood samples (n = 738) by conventional techniques using the Puregene® extraction kit (Gentra Systems). Class I HLA-A, -B and -C allele typing was performed using High Resolution SSP (sequence-specific primer) A, B, and C UniTray typing kits, respectively. Any ambiguities were resolved using the Forensic Analytical sequencing kit and AmbiSolv™ when needed (Invitrogen). Class II HLA typing was performed with high resolution DRB1 SSP, DQA1 SSP, DQB1 SSP, DPA1 SSP, and DPB1 SSP Unitray® typing kits with the entire locus on a single tray (Invitrogen). PCR was followed by AmbiSolv™ when needed and analyzed using MatchTools software. All reactions were run with negative controls, and every fiftieth PCR reaction was repeated for quality control. DNA samples were also genotyped for candidate SNPs selected from immune response genes using a custom designed 768-plex Illumina GoldenGate™ assay (Illumina Inc., San Diego, CA) along with SNPs selected from LTA (rs2857602, rs2857708, rs915654, rs2844482, rs1041981, rs1799964, rs1799724), TNF (rs1800629) and LST1 (rs2256965, rs2256974) gene families. Not all LTA, TNF and LST1 SNPs were genotyped due to different selection strategies and lack of multiplexing abilities in the genotyping system. SNP-specific deviation from Hardy-Weinberg Equilibrium (HWE) was tested and no LTA, TNF or LST1 SNPs displayed violations of HWE (*p*<0.001). Subject exclusions were made on the basis of DNA quality (n = 6), complete genotyping failure (n = 4) and low call rates below 95% (n = 14), leaving a total of 714 subjects in the study.

### Cytokine Assays

Cytokine IFN-γ, IL-2, IL-6, TNF-α, and GM-CSF secretion levels in response to rubella virus stimulation (W-Therien strain, gift from Dr. Teryl Frey, Georgia State University) were determined in PBMC culture supernatants by ELISA. We used previously optimized conditions specific for each cytokine in terms of the time of incubation and amount of virus [multiplicity of infection (MOI)] [21]. We quantitatively determined rubella-specific cytokine responses in cell-free supernatants by ELISA following the manufacturer's protocol (BD Biosciences Pharmingen, San Diego, CA). For all cytokine outcomes, we obtained three rubella virus-stimulated measures and three unstimulated measures. We subtracted the median background levels from unstimulated control cell cultures from the median rubella-induced responses to calculate corrected secretion values.

### Statistical Methods

We examined the following outcomes: a measure of circulating rubella IgG antibodies (measured in units of IU/ml) and five measures of rubella virus-specific *in vitro* cytokine secretion (IFN-γ, IL-2, IL-6, TNF-α, and GM-CSF, each measured in units of pg/ml). Per manufacturer specifications, antibody level assays resulted in one observation per subject. In contrast, assessment of cytokine secretion resulted in six measures per individual: three unstimulated and three rubella virus-stimulated measures. In order to describe these outcomes, a single value per individual was obtained by subtracting the medians of the three unstimulated values from the median of the three stimulated values. Data were descriptively summarized across individuals using frequencies and percentages for all categorical variables, and medians and interquartile ranges (IQRs) for continuous variables. Extended haplotypes were constructed using the three HLA class I loci (A, C, and B); seven common SNPs from the LTA gene (rs2857602, rs2857708, rs915654, rs2844482, rs1041981, rs1799964, rs1799724); TNF SNP rs1800629; two SNPs from the LST1 gene (rs2256965, rs2256974); and five HLA class II loci (DRB1, DQA1, DQB1, DPA1, and DPB1) (**Figure 1**). Design variables were created for individual haplotypes assuming an ordinal effect on immune response. Haplotype frequencies based on SNP alleles and two-digit HLA alleles were created using a maximum likelihood approach. Three sets of extended haplotypes were considered: one containing the three HLA class I loci and the set of SNPs, one containing the class II loci and the candidate SNPs and, and one containing both class I and class II loci with the set of SNPs. Because pedigree data were unavailable and each individual's linkage phase is unknown, there may be multiple pairs of haplotypes which are consistent with the observed genotypes. Posterior probabilities of all possible haplotypes for an individual, conditional on the observed genotypes, were estimated using an expectation-maximization (EM) algorithm implemented in the Haplo.Stats package [31], using the default setting for batch size, maximum number of iterations and convergence criteria. These posterior probabilities were used to construct an expected design matrix that contained as variables the expected number of copies of each haplotype carried by each individual. These variables in the expected design matrix range from 0 to 2, and make it possible to test for haplotype-outcome associations while accounting for phase ambiguity. Because of the imprecision involved in estimating the effects of low-frequency haplotypes, we retained only those occurring in our entire cohort with an estimated frequency of greater than 1%.

Formal associations of the six different immune response levels with the haplotype design variables were evaluated using linear regression models. Simple linear models were used to examine associations with antibody levels, whereas repeated measures approaches were implemented for the cytokine secretion variables. The repeated measures method improved statistical efficiency by enabling analyses that simultaneously included all six observed measurements. To assess haplotype associations with *in vitro* changes in cytokine immune response from an unstimulated to stimulated environment within this repeated measures framework, we included in the statistical model the design variable(s) of interest, an indicator of stimulation status, and the resulting interaction term(s). The strength of association was then formally tested by examining the statistical significance of the interaction term(s). We accounted for the likelihood of intra-subject correlations of immune response values by modeling an unstructured variance-covariance matrix within subjects.

Differences in immune response associated with a given set of common extended haplotypes were first simultaneously tested for statistical significance. This was accomplished by including all haplotype design variables with an estimated frequency greater than 1% in the linear regression model and assessing their combined effect on the immune response outcome using a multiple degree-of-freedom test. We estimated the proportion of variability in the immune response outcome attributable to the set of haplotypes by calculating the generalized R^2^ statistic which compared models with and without the haplotype design variables [32]. Following these global tests, we examined individual haplotype effects. For these tests, each design variable - and for the cytokine secretion data, stimulation status and the corresponding interaction term - was included in a separate linear regression analysis, effectively comparing immune response levels for the haplotype of interest against all others combined. This series of tests was performed in the spirit of Fisher's protected least significant difference test; individual associations were not considered statistically significant in the absence of global significance. Due to phase ambiguity, haplotype-specific descriptive summaries using medians and inter-quartile ranges could not be obtained for secreted cytokines. Instead, t-statistics were calculated reflecting the direction, and relative magnitude, of the estimated haplotypic effect on the immune response.

In order to assess the excess contribution of the SNPs on haplotype associations, over and above the HLA effects, we reconstructed and retested the haplotypes using only the HLA alleles. This resulted in three reduced haplotypes: one containing only the class I alleles, one containing only the class II alleles, and one containing both class I and class II alleles. All analyses described above were performed while adjusting for covariates potentially associated with immune response. The following set of variables were included in all models: age at enrollment, race, gender, age at first rubella vaccination, age at second rubella vaccination, and cohort status (first versus second). Data transformations were used to correct for data skewness in all linear regression models. We used a simple log-transformation for antibody level data. This approach was not feasible for the cytokine secretion data due to the existence of zero or negative values. Thus, for these variables an inverse cumulative normal (probit) transformation was used. All statistical tests were two-sided, and all analyses were carried out using SAS (SAS Institute, Inc., Cary, NC) and the Haplo.Stats software package as implemented in S-Plus (Insightful Corp., Seattle, WA).
